# The first use of a photogrammetry drone to estimate population abundance and predict age structure of threatened Sumatran elephants

**DOI:** 10.1038/s41598-023-48635-y

**Published:** 2023-12-03

**Authors:** Dede Aulia Rahman, Riki Herliansyah, Beginer Subhan, Donal Hutasoit, Muhammad Ali Imron, Didik Bangkit Kurniawan, Teguh Sriyanto, Raden Danang Wijayanto, Muhammad Hilal Fikriansyah, Ahmad Faisal Siregar, Nyoto Santoso

**Affiliations:** 1 Department of Forest Resources Conservation and Ecotourism, Faculty of Forestry and Environment, IPB University, Bogor, 16680 Indonesia; 2 Primate Research Center, Institute of Research and Community Service, IPB University, Bogor, 16151 Indonesia; 3 School of Statistics, Kalimantan Institute of Technology, Balikpapan, 76127 Indonesia; 4 School of Mathematics and Maxwell Institute for Mathematical Sciences, University of Edinburgh, Edinburgh, EH9 3FD UK; 5 Department of Marine Science and Technology, Faculty of Fisheries and Marine Science, IPB University, Bogor, 16680 Indonesia; 6 Jambi Natural Resources Conservation Agency, Jambi, 36361 Indonesia; 7 Tropical Biodiversity Conservation Program, Faculty of Forestry and Environment, IPB University, Bogor, 16680 Indonesia; 8 Yogyakarta Natural Resources Conservation Agency, D.I. Yogyakarta, 55514 Indonesia; 9 Frankfurt Zoological Society, Jambi, 36361 Indonesia

**Keywords:** Biological techniques, Biotechnology, Ecology, Zoology, Ecology

## Abstract

Wildlife monitoring in tropical rainforests poses additional challenges due to species often being elusive, cryptic, faintly colored, and preferring concealable, or difficult to access habitats. Unmanned aerial vehicles (UAVs) prove promising for wildlife surveys in different ecosystems in tropical forests and can be crucial in conserving inaccessible biodiverse areas and their associated species. Traditional surveys that involve infiltrating animal habitats could adversely affect the habits and behavior of elusive and cryptic species in response to human presence. Moreover, collecting data through traditional surveys to simultaneously estimate the abundance and demographic rates of communities of species is often prohibitively time-intensive and expensive. This study assesses the scope of drones to non-invasively access the Bukit Tigapuluh Landscape (BTL) in Riau-Jambi, Indonesia, and detect individual elephants of interest. A rotary-wing quadcopter with a vision-based sensor was tested to estimate the elephant population size and age structure. We developed hierarchical modeling and deep learning CNN to estimate elephant abundance and age structure. Drones successfully observed 96 distinct individuals at 8 locations out of 11 sampling areas. We obtained an estimate of the elephant population of 151 individuals (95% CI [124, 179]) within the study area and predicted more adult animals than subadults and juvenile individuals in the population. Our calculations may serve as a vital spark for innovation for future UAV survey designs in large areas with complex topographies while reducing operational effort.

## Introduction

The Indonesian Island of Sumatra has one of the highest rates of deforestation in the world as a result of a variety of anthropogenic activities, including (1) the conversion of forests for industrial plantations, (2) semi-forest fires, (3) small-scale forest clearing, and (4) road construction^1,2^. A total of 16.2 million ha of Sumatra's primary forest has been destroyed since 2000–2012 at a conversion rate of roughly 2,857 ha (17.63% year^−1^)^1^. Most forest loss (> 80%) has occurred in lowland areas with easy access, the most diverse ecosystems and significant carbon stocks^3^. There are 13 orders of mammals known to inhabit Sumatran tropical rainforests, one of which includes the highly renowned and endangered Sumatran elephant (*Elephas maximus sumatranus*). The species is protected under Indonesian laws^4^ and categorized as Critically Endangered on the IUCN Red List^5^. Based on data compiled by the Indonesian Elephant Conservation Forum (FKGI) in 2019, the total elephant population in Sumatra, is estimated 924–1359 individuals^6^.

In order to effectively manage wildlife conservation efforts, it is crucial to have frequent, precise, and reliable information on the presence, population structure, and density of a species across its entire range. For various terrestrial mammal species, data on species' presence, structure, and population density have been traditionally collected by observing and counting individuals along line transects by foot^7,8^ during general biodiversity^9^ or species-specific surveys^10^ together with camera trap surveys^11,12^. Population structures, i.e., age composition, sex ratio, growth rate, and survival rate, of wild animals are rarely known accurately. They are often estimated through direct observation of body size or the development of specific animal characteristics (e.g., skin color or growth of cheek pads in male orangutans)^13^. However, this method is often subjective and relies heavily on the researcher's experience and judgment. As an alternative, scientists have begun measuring specific body parts and comparing them with other indicators to more accurately determine the animal's age (e.g., the structure of the teeth of elephants^14^). Obtaining accurate morphometric data on wild animals is possible by directly measuring their body size in the field, however this requires individuals to be captured and chemically immobilised^15,16^. This method is invasive and not recommended for some species because it carries a high risk of stress and death^17^. Total body length is regularly used, given there is often a well-known, strong relationship between this variable and age^18–22^. In previous studies using an invasive approach, eye lens size, tooth eruption and wear patterns, tusk length, body weight, chest circumference, body length, and body height have been used to differentiate elephants into several age classes^14^. However, most invasive approaches used in anatomical studies (e.g., tooth eruption) are not feasible in field studies.

Accurately determining the age, sex, and population size of wild Sumatran elephants pose a challenge due to limited knowledge of effective and reliable methods, despite various techniques available to determine population demographics, such as dung and DNA surveys^23^. The difficulty arises from the high degree of fission–fusion dynamics displayed by Sumatran elephants, where group members split into subgroups with varying sizes, membership, and spatial cohesion over time^24^. In addition, these species have subtle sexual dimorphism, have large home ranges, and move in areas with high tree density, which can make them difficult to survey and may result in unsurveyed populations in certain locations^25^. Additionally, the wide distribution of the species and the costs of conducting ground surveys further hinder factors^26^. Rapid changes in land use due to various anthropogenic activities have the potential to cause changes in Sumatran elephant population dynamics over time, which will determine the sustainability of the species. Hence, frequent surveys and monitoring are required to obtain data on population density and demographics at a pace comparable to land-use change. Realizing this requires the urgent testing of alternative, non-invasive methods.

Sumatran elephants are often elusive, widely dispersed, and commonly occur in complex environments or inhabit inaccessible areas, which increases the potential for imperfect detection and false detections during counts^27^. For cryptic, widely dispersed species, N-mixture models, in which repeated counts at survey sites are used to estimate both probabilities of detection and population size, are often considered the most useful and practical^28,29^. This is because they are the only repeat visit approach that allows abundance estimation while accounting for imperfect detection, a challenging and costly task for elusive species, particularly if they occur in private or inaccessible areas^28^. Therefore, N-mixture modeling is less time, cost- and labour-intensive than other repeat visit approaches, estimates can be made over larger areas, and the approach is suitable for protected species^30^.

Photogrammetry has recently been considered a viable alternative non-invasive approach to collecting demographic data^20,31,32^. In photogrammetry, knowledge and technology are used to obtain reliable information about a physical object and its surroundings through recording, observing/measuring and interpreting photographic images^33^. However, this approach involves ground observers using a camera, which puts researchers close to the animal target, poses a significant risk to observers and may disturb target species. More recently, photogrammetry has been continuously developed using images collected from aerial surveys using drones^18,34,35^, taking advantage of the ability to capture animal data without being near the animals. Drones assist in pinpointing areas, monitoring behaviors and movements, and evaluating interactions between wildlife and their habitats^36^. In various studies, drones equipped with standard visual spectrum (RGB) cameras have been employed to gather information on the presence of species because of their capacity to cover vast territories expeditiously. Recently, drones have also been utilized to calculate population density^20^. There is a known relationship between body length and lifespan of wildlife^37–41^; using UAV, i.e., drone photogrammetry, that can capture images of wildlife, and the Sumatran elephant is a promising subject for developing rapid, accurate, and non-invasive methods to estimate population abundance and determining its age and sex.

Accounting for the potential of imperfect detection and false detections during drone surveys and the difficulty in obtaining total body length measurements of Sumatran elephants, the aim of this study was (1) to estimate the population abundance of a Sumatran elephant using N-mixture models from drone data derived RGB imagery and automated detection and (2) to establish a more reliable method to age Sumatran elephants by using body length measurements on photogrammetry from drone imagery and statistical analysis. Our study used statistical analyses to predict age and population structure in Sumatran elephants using drone photogrammetry, with results validated using morphometric data from a zoo to produce a reliable and robust age estimation method for the Sumatran elephant population.

## Results

### Estimation of population size

We present the posterior summary statistics of three parameters of interest: ${N}_{total}$ (the abundance estimate), $\lambda$ (the expected population size at each location) and $\psi$ (the detection probability) in Table 1. The trace plot in Fig. S1 and the $R$ statistic (< 1.10) in Table 1 for each parameter indicates no lack of convergence, thus we may proceed to the inference on abundance estimation and detection probability.

**Table 1:** Posterior summary statistics of N-mixture model parameters fitted on the elephant count data, corresponding to the posterior mean, standard deviation (SD), median, the 95% lower and upper credible interval (LCL, UCL) and the $R$ statistics.

Parameter	Mean	SD	Median	LCL	UCL	$R$
$\psi$	0.55	0.06	0.55	0.44	0.65	1.00
$\lambda$	19.01	2.41	18.83	14.45	23.74	1.00
${N}_{total}$	151.16	14.79	149	124	179	1.01

The model was fitted using a Bayesian approach for 50,000 iterations with priors: $\psi \sim {\text{Beta}}\left(\mathrm{1,1}\right)$, $\lambda \sim {\text{Uniform}}\left(\mathrm{0,100}\right)$, and ${N}_{j}\sim {\text{Poisson}}\left(\uplambda \right)$.

We obtain the estimate of elephant abundance of 151 individuals within the study area with 95% credible interval $\left[124, 179\right]$. The expected population size $\lambda$ is estimated to be approximately 19 individuals per location and remain the same over 8 locations with 95% credible interval $\left[14, 24\right]$. This estimate implies that we expect populations of approximately between 14 and 24 individuals which are available for sampling at each location. Finally, we obtain the estimate of detection probability at each location $\psi$ of $0.55$ with 95% credible interval $\left[0.44, 0.65\right]$. The probability of 0.55 indicates that we may detect approximately 55% of the population at each location i.e., over half of the expected population. Note that the detection probability may vary within the locations depending on the geographical or environmental conditions, but within this study, we do not consider such variability.

### Age prediction

We applied models to the elephant dataset with 23 known-age individuals from a zoo and obtained parameter estimates. Predictions of age based on these parameter estimates for each model, involving interpolated body lengths, produced prediction intervals (Table S1). Due to the high uncertainties in the von Bertalanffy model e.g., wide prediction intervals, we prioritize the GAM for further analysis. The GAM achieved an adjusted ${R}^{2}$ of 0.89 and explained 92.6% of the deviance, indicating a good fit. Figure S2 shows the partial effect of body length on the smooth function, $s\left({L}_{i}\right)$, indicating a positive effect of body length, particularly at lower values, with a non-linear decrease as body length approaches the asymptotic body length estimated from the growth model (${L}_{\infty }$ = 512).

**Figure 1:**
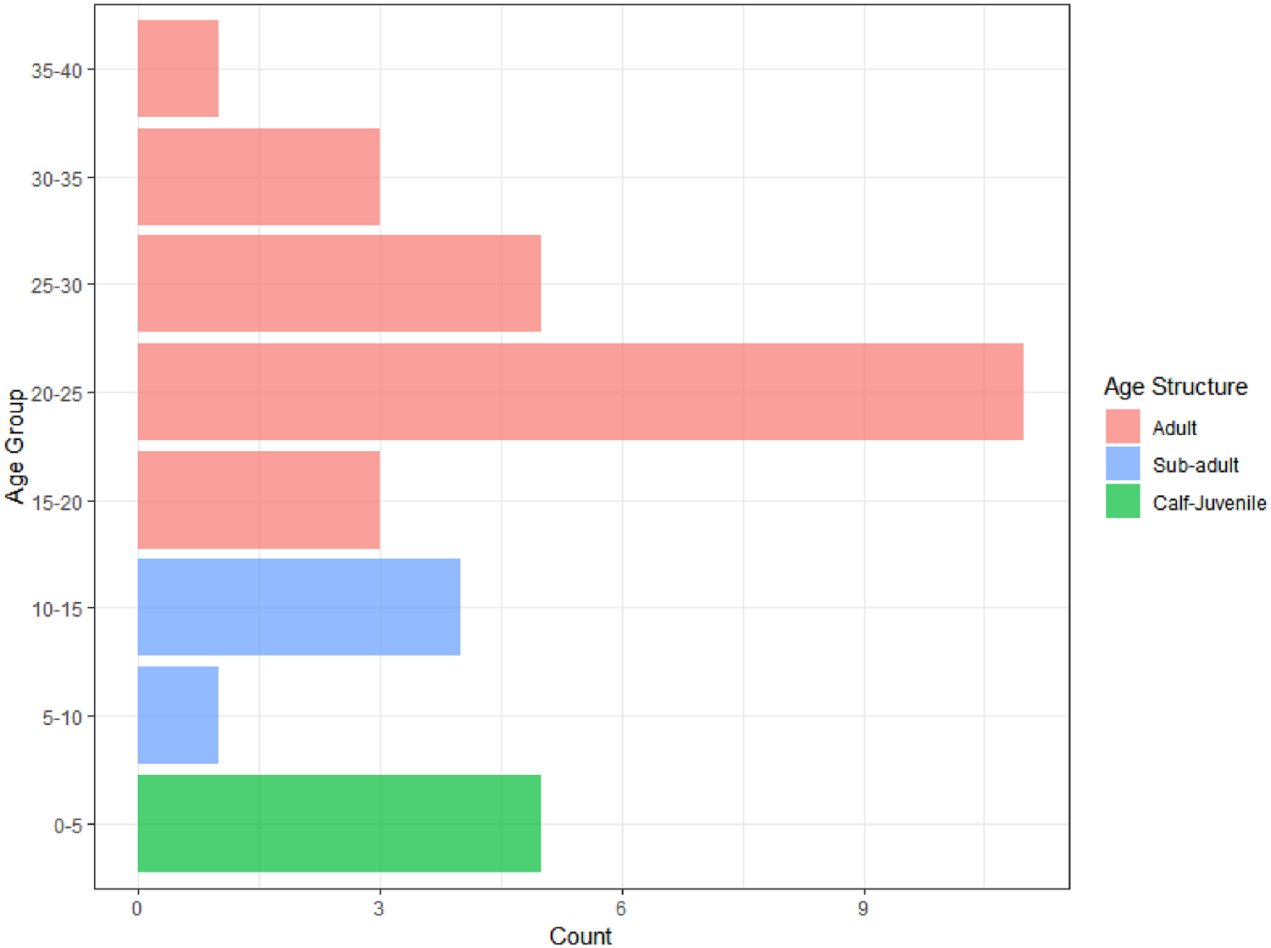
Age distribution estimated using GAM of wild elephants (n = 33) found during the survey.

Our next step involves age predictions for elephants based on aerial survey data. A dataset comprising imagery of thirty-three individual elephants across eight distinct locations was collected. Total body length calculations for each image were performed as a means of estimating the age of elephants. The calculated body length values span a range from 140 to 325 cm, which aligns with the range encompassed by the model-fitting dataset. We create an age group for predicted values from the GAM model according to: (0, 5), (5, 10), …, (35, 40) to aid the interpretation. Figure 1 illustrates the age structure of the thirty-three wild elephants, categorized into eight distinct classes. Notably, the highest concentration of individuals is observed within the age group of 20–25 years, representing the adult population that shows the skewed age structure of the population.

## Discussion

**Figure 2:**
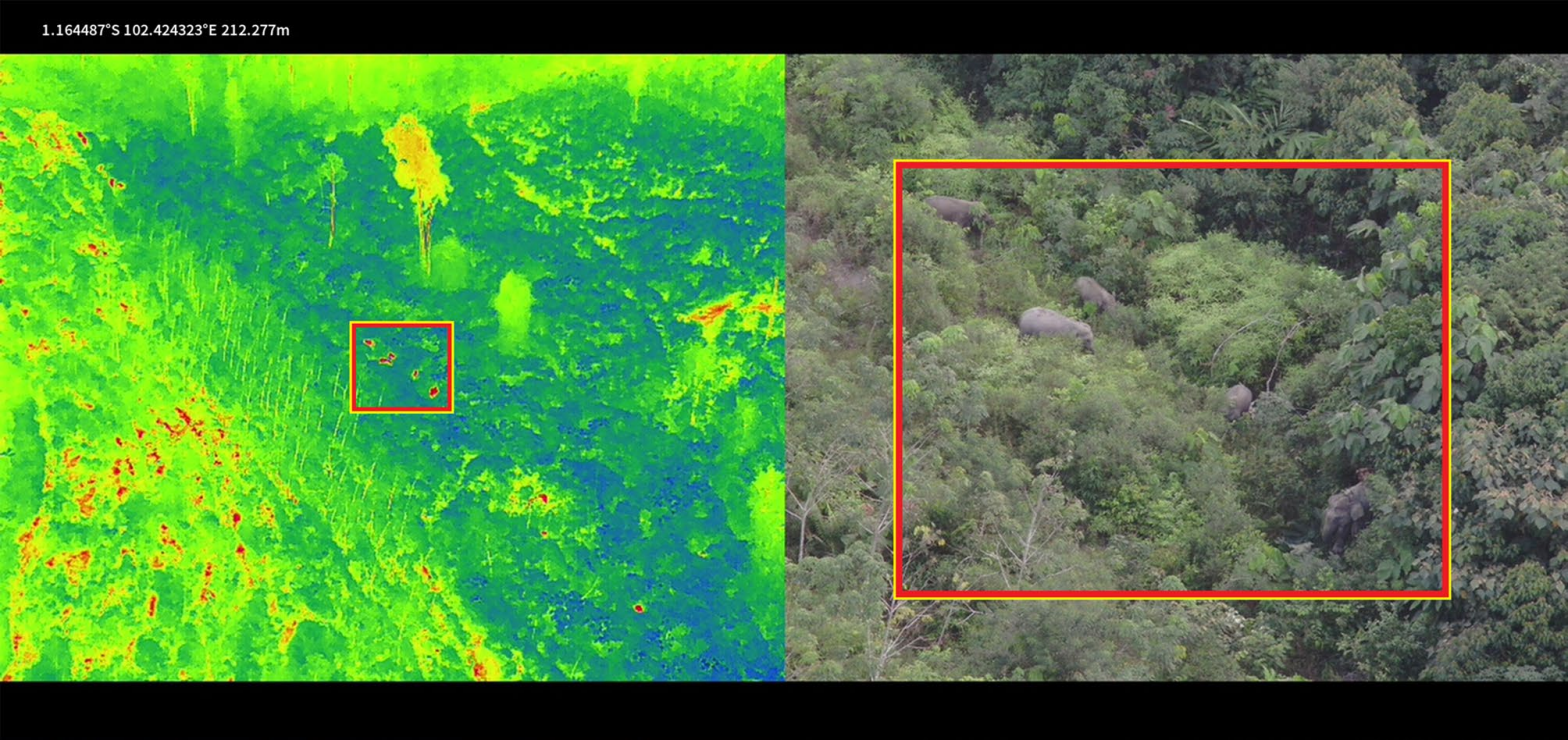
Sumatran elephant on RGB imagery confirmed via TIR imagery on drone flights in August 2023 at 100–120 m altitude.

Obtaining accurate calculations using conventional survey methods in tropical rainforests is challenging due to the need for well-trained field workers and long survey durations to cover large areas with challenging terrain. Detecting animals in tropical rainforests can be difficult due to weather conditions, dense and hard-to-reach canopies, and the visual camouflage of various species^42,43^. It is crucial to develop a method that can improve the accuracy, precision, and bias reduction in Sumatran elephant surveys. It has been acknowledged that drones have numerous benefits over traditional techniques^44–46^, and our recent research further validates the effectiveness of drone surveillance, especially in monitoring endangered biodiversity in remote areas that are rapidly experiencing land use change and disturbance. In recent studies, drones have been recognized as an innovative and valuable tool for conducting conservation research on many species across different types of ecosystems in Indonesian tropical rainforests, for example, drones for biodiversity surveys^36,47,48^, studying primate behaviour and waterbird population survey^36^, and we confirm the feasibility of this method for conducting non-invasive aerial surveys on the Sumatran elephant. This species is ideal for drone monitoring because it behaves and lives in groups—congregating in relatively open forest areas to feed and shelter under forest stands—and is highly visible from the air. Also, they have a group defence system when they feel threatened by large carnivores and humans, which could potentially cause harm to humans who are near them. In addition, we observed that Sumatran elephants are less disturbed by drones than ther are by humans, resulting in fewer individuals hiding during aerial surveys, in accordance with studies in other remote areas^36^. The effect is especially true for smaller individuals and areas where threats by large carnivore species and humans are common, and groups of elephants tend to be more agitated^49^. Another crucial factor to consider is that drones can prevent "convenience sampling"^50^ by providing easier access to remote areas, allowing for comprehensive surveys of the entire potential distribution of known species with a greater chance of discovering unreported or elusive groups. Our firsthand experience validates this, as we were able to locate individuals or groups more efficiently through aerial surveys and with the assistance of the thermal sensors on the drones we employ (Fig. 2).

We successfully tested the suitability of drones and N-mixture models for monitoring the population status of the Sumatran elephant in its tropical forest habitat. The combination of multi-sensor cameras and Global Positioning System telemetry in our study could improve the accuracy of estimates for performing N-mixture models and analysing how species’ home ranges can affect the required size of the area sampled. Estimates obtained with the N-mixture models were more precise (narrower confidence intervals) but the numbers are not significantly different than those obtained using non-invasively collected DNA-based capture-recapture methods in previous research^23^. Moreover, the survey time is much shorter using drones and does not require comprehensive laboratory knowledge with expensive costs for collection and data analysis. Our population estimates are comparable to previous studies, indicating that drone surveys have the potential to continue to be developed and used to monitor multiple wildlife species in tropical forest areas. However, the elephant avoidance and thick canopy forest may present challenges in capturing the target species using drones and may potentially lead to bias in estimation. Therefore, we may need to increase the search effort and sampling areas, especially when no prior information on individuals is available. We also note that a sensitivity analysis of selecting a given sample area is suggested as a future direction of this research for the evaluation of its impact on the N-mixture model parameters^51^. Furthermore, the reliability of the abundance estimates can be improved by incorporating external information e.g., information on elephant abundance from different sources, to construct informative prior on $N$ for Bayesian analysis. Within the scope of Sumatran elephant conservation throughout its native range, the use of drones as a monitoring tool should be encouraged to rapidly and accurately collect current data on elephant populations in 23 known elephant habitats^52^. In the past, there have been varying estimates of the elephant population in the BTL, which includes the Sumai and Riau-Jambi areas. Those estimates ranged from 300 to 400 individuals in 1984^53^ to only 50 in 2007^1^. However, with the exception of a dung count census from 2009^54^ that estimated 117 elephants for the Sumai area and 47 elephants for the Riau-Jambi area, available estimates are either outdated, entirely based on guesswork, or both, and therefore do not likely reflect the current population size. While^23^ abundance estimates were very similar to those of the dung count from 2009^54^, being the most recent and most precise study, our estimates of 151 individuals within the study area (95% CI [124, 179]) should be used for conservation planning and as a baseline for monitoring. It is important to note that the low elephant densities in the study site might be due to patchy habitat usage and increased elephant mortality caused by human activities. This assumption is supported by the skewed age structure of the population and evidence of elephant killing in the past. Although direct evidence is lacking, elephant killings were likely more frequent before 2012, as the area was largely uncontrolled and unmonitored. Therefore, we can assume that human activities have influenced the population structure in BTL, as has been observed in other elephant populations in Asia^55,56^.

Differentiating the age categories of the Sumatran elephant can be challenging due to their activity patterns, which are often covered by a dense forest canopy, and the subtle sexual dimorphism of the species. Total body length is a reliable indicator of age, as it often correlates strongly with the animal's age^18,21^. However, obtaining the necessary body measurements from images is not always feasible, particularly if the animal is partially obscured. There are various approaches to handling missing data. Usually, animals without the required measurements are excluded from further analysis^57^, or replaced with the average measurements of other individuals whose ages are confirmed, as we did in our study, where we utilized data on individual elephants in zoos to develop the initial model^58^. Our research shows that GAM is quite precise in predicting the age of elephants, which may be due to the similarity in the relationship between age and length in the inverse of von Bertalanffy's growth model to GAM. In particular, the von Bertalanffy model specifies the predictor as a function of the asymptotic size (${L}_{\infty }$) and the body length (${L}_{i}$)^59^, while GAM models the predictor as a smooth function of unknown parameters and seeks the best fitting line to the data, e.g., minimum prediction errors^60^. However, we note that GAM is a data-driven model where the quality of the model, e.g., precision, depends on the data, and the associated parameters do not have a biological interpretation, like growth parameters in the von Bertalanffy model. Thus, GAM is not an appropriate model when the objective of the study is to obtain and understand such growth parameters but is a good model for predictive purpose. We further note that age prediction is feasible when prior information is available to build the models e.g., von Bertalanffy and GAM, thus requiring measurement data of captive individuals. When such data is not available, past research on growth parameters may still be utilized for prediction (e.g.,^61^). The increasing number, class width narrows and natural variation in length may result in greater overlap of length between age classes, but the information obtained is still useful as can robustly discriminate between old and young individuals e.g., juvenile, sub-adults and adults. Finally, we note that sex characteristics may affect the age prediction due to their difference in body lengths^62^. To account for such variability, the model can be separated into two and obtain the estimate for each sex or the sex indicator may be included as a predictor (only feasible for GAM).

Asian elephants generally live longer than African elephants and are slow reproductive animals with a relatively high survival rate to old age after a slightly riskier postnatal year^55^. In natural populations in BTL, we found more adult animals than subadults and juveniles, as observed, inter alia, by^63^ and^64^ in India. Our sample, however, compared with the survey results of^23^ in 2011, and revealed an increasingly favourable age structure for the population in Bukit Tigapuluh, with an almost larger number of adults than other age classes. The adult sex ratio (ASR) of a population is a critical parameter in ecology due to its expected impact on demography and opportunities for selection. Biased ASR can lower reproduction or survival rates for the less abundant sex^65,66^. This can substantially reduce population growth and even lead to collapse^66^. Moreover, we found 2 to 3 calves per group of elephants. However, it should be noted that most elephant deaths due to conflict and poisoning in response to crop raids, occur in adult individuals. Based on computer simulations by^55^, the population suffering from increased adult mortality shows a relatively increased number of calves, shifting the general age distribution to a younger class. Thus, a young population does not necessarily mean recovery, but could instead be a sign of heavy losses in the older age classes.

In the management of the Sumatran elephant population, drones can provide informative observations and be seamlessly integrated into current monitoring practices. With low-cost drones, orthomosaic collection is easily replicable and produces high-resolution mosaics that enable the study of population demographic and spatial location of the elephants. This information is crucial for managing elephant populations and their habitat in BTL, particularly since most elephants reside outside protected areas. Orthomosaics that include the location of individual elephants can reveal how they are distributed across the landscape, habitat condition, and the effects of human activity. This automated workflow can be used to assess the demographics and condition of populations of Sumatran elephants and other large mammals by increasing statistical power, and providing new tools for research and population management. Moreover, with advanced machine learning algorithms for data processing, the photogrammetry from drones might also reveal insights into population health or age and other parameters, which offers exciting opportunities.

**Figure 3:**
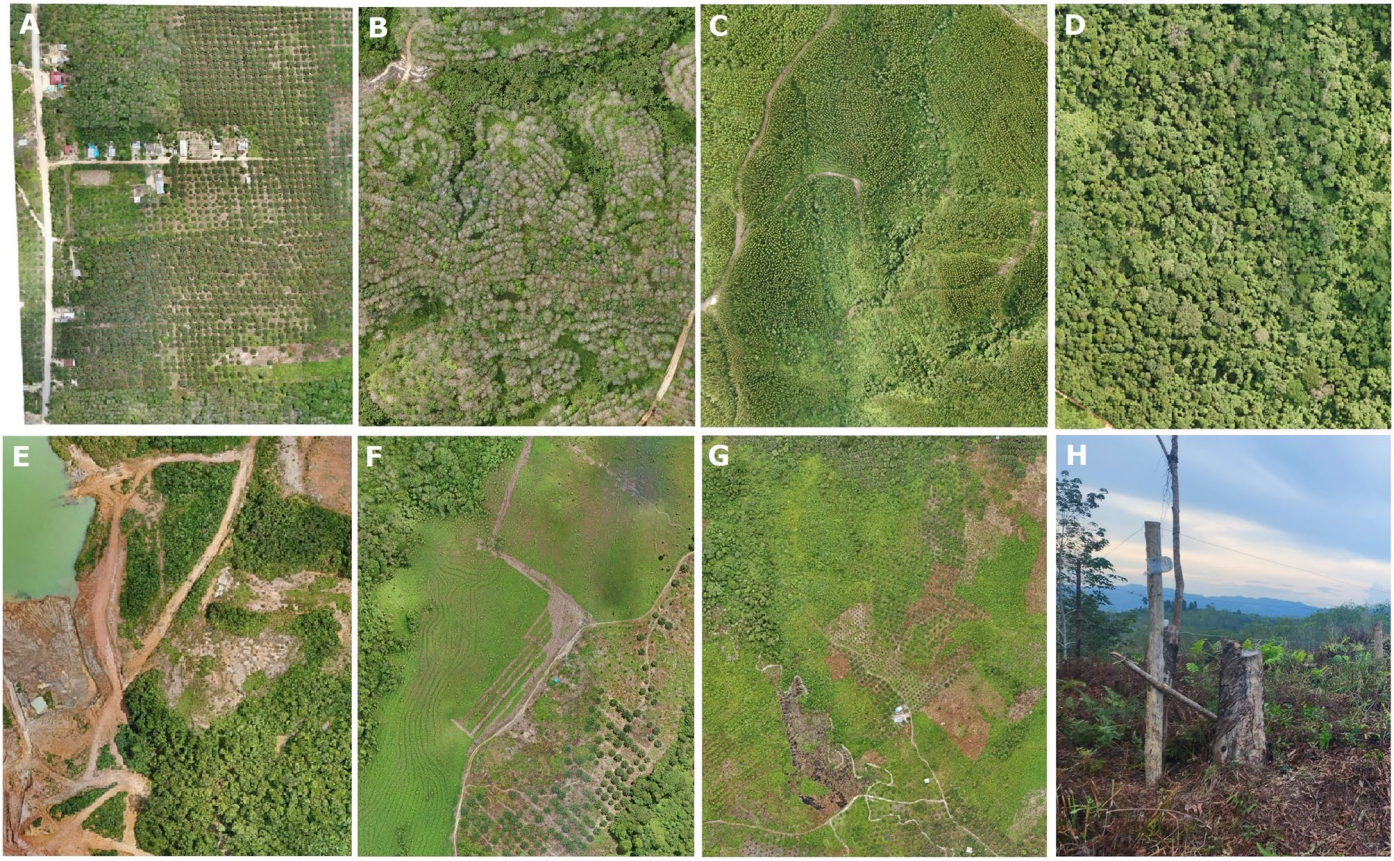
Land covers dominate the landscape: (**A**) oil palm plantations, (**B**) rubber plantations, (**C**) eucalyptus plantations, (**D**) secondary forests, (**E**) coal mines, (**F**) open areas, (**G**) land clearing for agriculture land, and (**H**) electric fence along the oil palm plantations area.

The main concerns in the BTL, which drone surveys have monitored, are land conversion, deforestation and massive and uncontrolled installation of electric fences, leading to human-elephant conflict, poaching, and a decline in Sumatran elephants' quality of life and restrictions on their movements (Fig. 3). This situation has the potential to increase the death rate of elephants beyond their birth rate. While drones can offer tailor-made solutions for wildlife conservation management problems, the social implications must also be considered^67^, especially in areas such as the BTL, where conservation decisions are very sensitive and often affect human lives and livelihoods. Previous studies and our drone monitoring of the BTL indicate that most of the elephant’s roam outside protected areas. Illegal clearing of forests by communities to support their livelihoods through farming in the southwestern of BTL must be controlled, and strong law enforcement is needed to halt destructive practices. The co-existence of elephants and humans on the intensively used agricultural lands of the BTL may be improbable, but the extensive production forest concessions surrounding Bukit Tigapuluh National Park provide a potentially safe habitat. Within these concessions, wildlife-friendly management outside of statutory conservation commitments can convert large parts of the landscape into suitable elephant habitats without eliminating all commercial interests. Conservation of the elephant population and their habitat in this region should thus be a high priority. Conservation management is more of a political decision. However, close monitoring can be crucial in judicious decision-making to support conservation, particularly in conflicting human interests. Technology can assist, supplement and strengthen the exercise for that purpose.

Our study shows that drones have become a powerful tool in monitoring and mapping changes in species and disturbed ecological landscapes, offering a cost-effective and efficient way to access hard-to-reach areas and observe subtle environmental changes. This paper demonstrates that drone-acquired images can be processed to generate valuable data layers that can provide insights into species and landscape conservation and inform restoration and management efforts, which can be adapted to other regions. Drone surveys combined with hierarchical modeling and machine learning to estimate population abundance and age structure could have wider applications for other species in other study systems. The demographic population data of species from programmed flights by drones, as the results of our study, enables tracking population trends that can be used to establish management priorities and guide the implementation of species conservation measures in the landscapes. The demographic population data from species enables tracking population trends that can be used to establish management priorities and guide the implementation of conservation measures in the landscapes.

## Methods

### Study area

**Figure 4:**
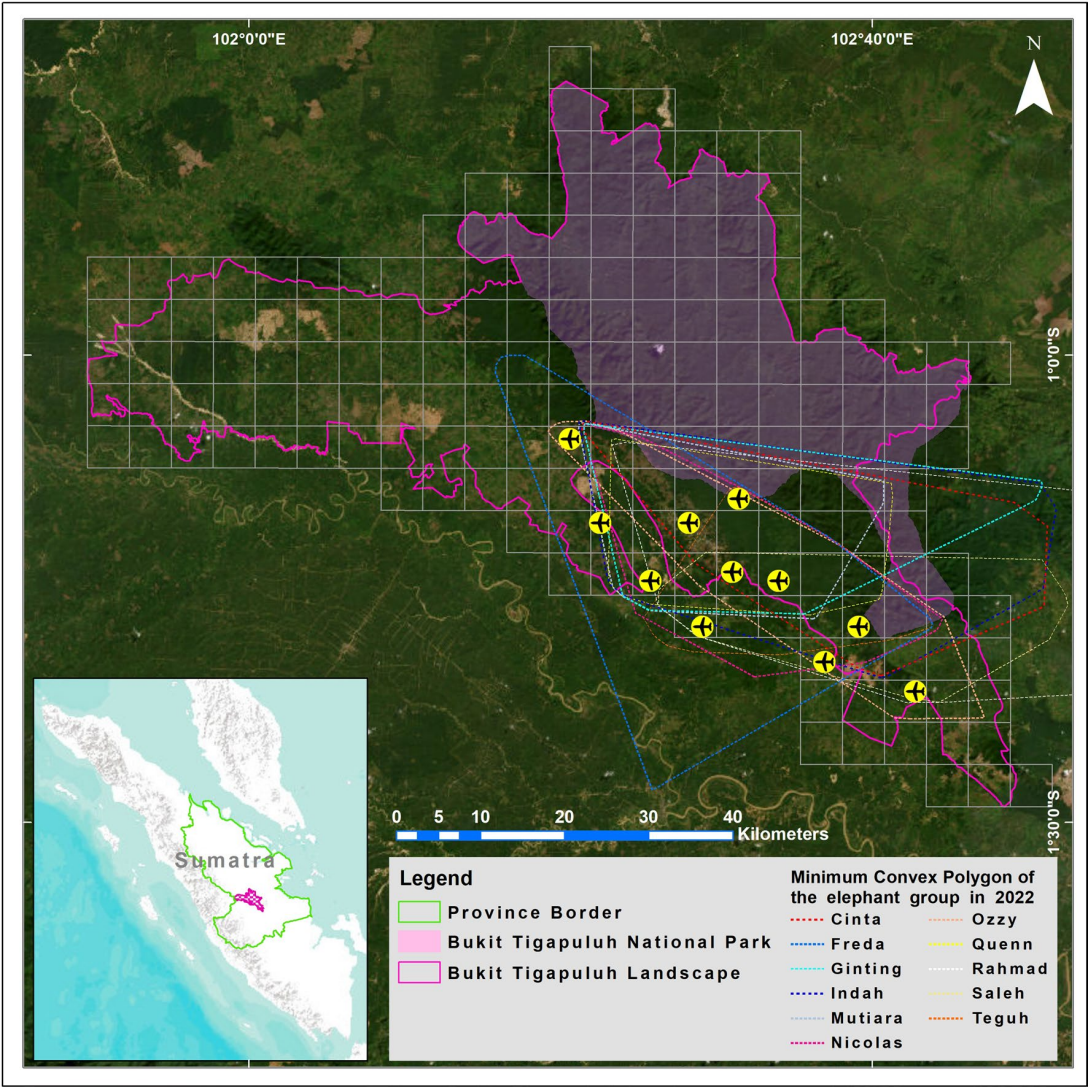
Study design of drone in Bukit Tigapuluh Landscape.

The Bukit Tigapuluh landscape (BTL) is situated in the centre of Sumatra Island (1°4′27.72ʺS and 102°30′43.89ʺE) and spans across two provinces, namely Riau and Jambi (Fig. 4). The area encompasses over 3500 km^2^ of land, including the Bukit Tigapuluh National Park (1440 km^2^) and is home to the Talang Mamak indigenous community^68^. The study area of BTL includes the Datuk Gedang Essential Ecosystem Area (KEE), which covers 618.29 km^2^. The original vegetation, composed of extremely species-rich dipterocarp rainforest^69^, is now largely limited to the rugged center of the landscape and surrounded by a patchwork of various land-use types, including oil palm, rubber tree plantations, and pulpwood plantations covering ± 75% of the total landscape, non-active former logging areas are now partly covered with secondary forest (17%), and surface coal mining areas, small settlements, and private farmland with the smallest proportion. Based on its function, Datuk Gedang is located in a cultivation area covering 457.12 km^2^ of Production Forest (PF), 157.97 km^2^ of Limited Production Forest (LPF) and 1.38 km^2^ of Other Uses Areas (OUA). The PF and LPF areas are industrial plantation forests of rubber and acacia trees which incorporate a social forestry area. The OUA is a residential area and a garden for the local community. Bukit Tigapuluh Landscape also has a concession in the form of an ecosystem restoration timber forest product utilization area. The climate is tropical, with warm temperatures throughout the year (mean annual temperature = 22 °C, min = 21 °C, max = 33 °C) and high rainfall (average rainfall = 2577 mm/year, max = 347 mm/month, min = 83 mm/month), and altitudes ranging between 60 and 843 m asl^68^.

### Drone surveys

Drone data was collected in November 2022 using a Dà-Jiāng Innovations (DJI) Matrice 300 RTK outfitted with a DJI Enterprise Zenmuse H20 Series multi-sensor camera. This camera includes a zoom, wide, and thermal camera (H20T), which utilizes an Uncooled Vanadium Oxide (Vox) Microbolom sensor for thermal detection. The drone has a maximum flight time of 30 min, a top speed of over 70 km h^−1^, a pilot-controlled range of more than 5 km, and a 20 MP camera capable of recording high-definition 4 K/60 fps video. We programmed all flight plans using Mission Planner (DJI Pilot version 1.9.0 by DJI Innovations Technology Co., Ltd., Shenzhen, China). All images were compiled and ortho-rectified using Agisoft Photoscan Pro ver. 1.2.5.2594, now known as Agisoft Metashape (https://www.agisoft.com/accessed as of 18 July 2023), before being imported into QGIS ver. 2.8.6 (QGis Development Team, Beaverton, OR, USA) for analysis.

The entire study area of 3500 km^2^ in the BTL was divided into 140 grids of 5 km × 5 km each. Previous survey data and information, such as the direct presence of elephants in the landscape and indirect encounters through traces of activity from the ongoing long-term research, local field guards, and forest officials were integrated into the spatial layers and overlayed in ArcGIS 10.6. Our study focuses on the central and southeastern parts of BTL since GPS collar data shows elephants are no longer moving southwest due to the development of agricultural land, illegal settlements, and the construction of uncontrolled electric fences that cut off the movement paths of Sumatran elephants. However, in principle, elephant surveys using drones can indeed be conducted without using GPS collars, but still consider preliminary surveys regarding the locations of elephant movement boundaries. Surveys of presence indicators such as dung, footprints, and information from local communities can determine the limits of elephant movement, which is very useful in optimizing resources and references in limiting survey locations. Like the transect and DNA surveys conducted by Moßbrucker in 2011 in the BTL, pre-surveys are crucial for successfully identifying the research boundaries to be undertaken. In preparation for implementing drone surveys to study Sumatran elephants, we conducted tests to assess any potential disturbances caused by the drones, such as evasive maneuvers or attacks, that could impact the accuracy of the survey results and minimise the disturbance of elephants. Based on our findings, we determined the minimum flying height of 100 m for the drones to avoid disturbing the elephants, which was the same height as their last flight before fleeing. We ensured that our drone's flying height did not exceed 120 m, which complies with the regulations outlined in the Regulation of the Minister of Transportation of the Republic of Indonesia No. 37/2020 regarding the Operation of Unmanned Aircraft in Airspace Served by the State of Indonesia.

**Figure 5:**
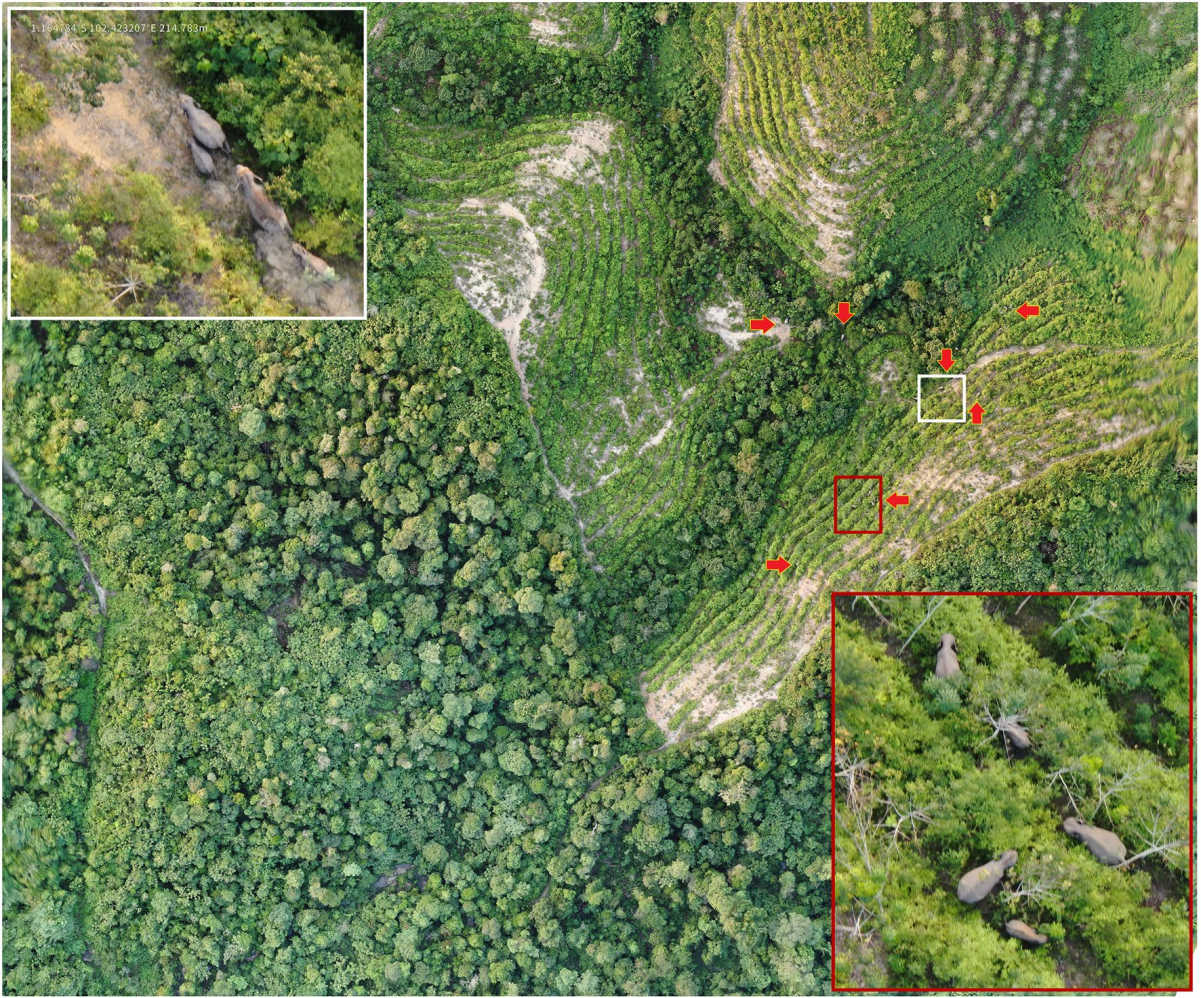
23 individual Sumatran elephants recorded in orthomosaic images and depicting the fusion between the Ginting and Indah groups in a rubber plantation.

Prior to conducting the drone survey, we verified the location coordinates acquired from the GPS collars. The collar information is used to cluster sample areas based on elephant movements throughout 2022 in BTL, thus narrowing the potential habitat of elephants. Moreover, the collar information is used to strengthen our assumption that elephants no longer move southwest for various reasons. The selection of survey locations is partially random, with a few referring to collar information. Before the drone flight is carried out, if we found any indication of the presence of elephants around the flight location from radio telemetry signals, we would launch the drone towards the sample location approximately 300–500 m from the GPS collar transmission point to avoid disturbing the elephants. Telemetry only functions as a reference for the elephant's location to avoid interference and not as a reference in choosing a drone flight location. The minimum altitude for each flight was set to 100 m. We conducted optimal flight evaluations and carried out drone surveys at an ideal speed rate of 5 m s^−1^, with a 90-degree camera orientation. The drone followed a pre-programmed flight plan autonomously, from take-off to landing. We conducted four flights at each site. The drone was programmed to take photos at regular intervals to ensure optimal photo collation and avoid shadows on maps, maintaining an 80% forward overlap and 80% lateral overlap between two consecutive images^36,47^. After conducting a field survey, we created maps and visually inspected them to detect the presence of the species (Fig. 5). We used photographs to capture each individual's head or body shape and record their body length. To create high-resolution images of each site, we combined approximately 100 images and corrected the perspective using Pix4Dmapper (v4.7, Pix4D SA). We obtained Ground Control Points (GCPs) from a Google Satellite online map to ensure accurate positioning matching with the satellite data. We then selected 10 GCPs on each orthomosaic to match the coordinates of the satellite image^70^.

### Automated detection and body size measurements of Sumatran elephant

**Figure 6:**
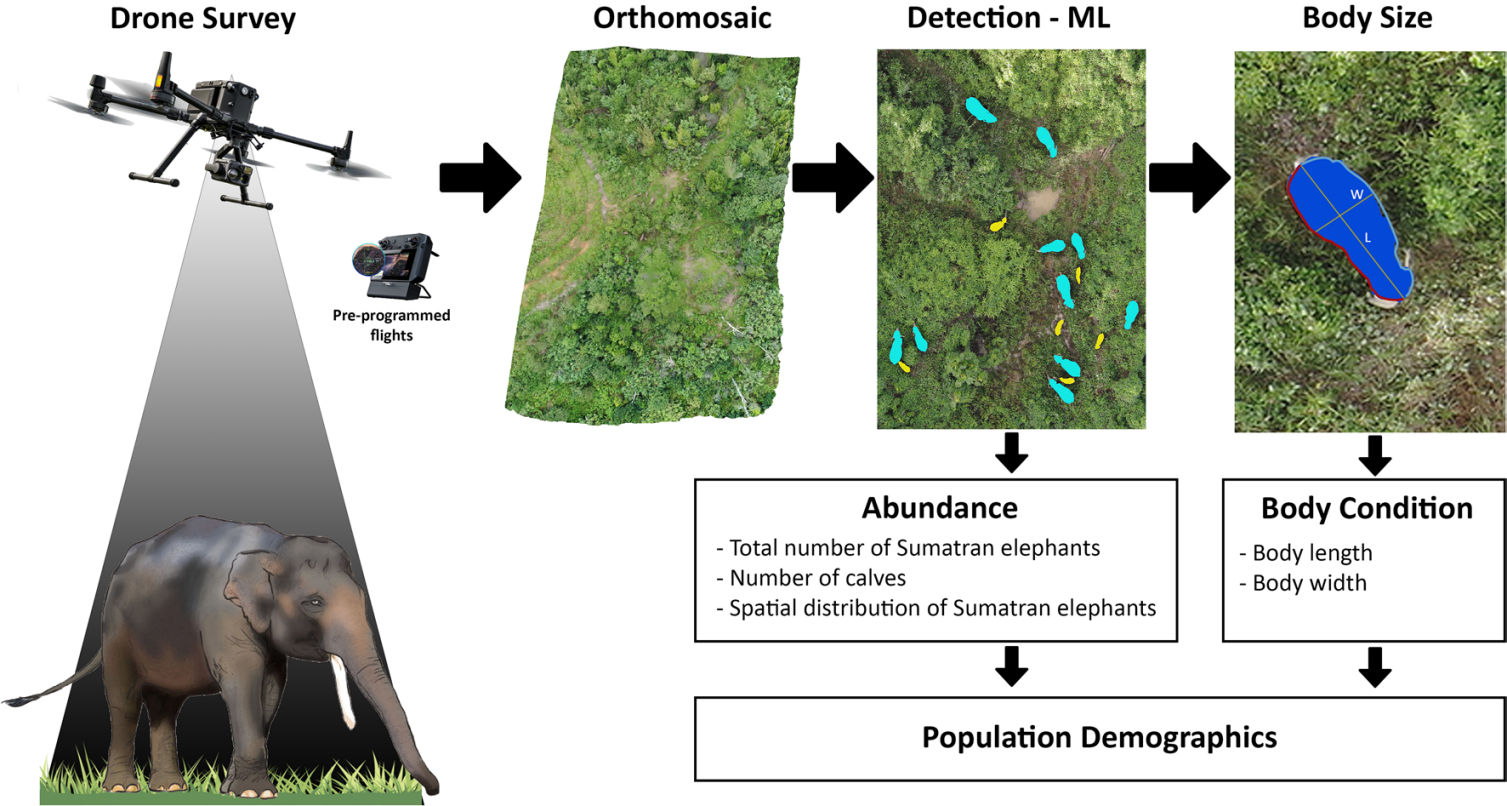
Overview of the automated work-flow for wildlife surveys, from drone flights to image analysis. Drones are used to collect hundreds of images that are converted into orthomosaics. Thereafter, animals are identified by machine learning and their body sizes measured.

To count the number of elephants accurately, we utilized Picterra, an online machine-learning (ML) platform that employs a Convolutional Neural Network (CNN) architecture for object segmentation. CNNs are advanced Deep Learning tools that can recognize and delineate specific object classes from raster images by analyzing patterns in pixel relationships. This method provides a reliable and automated alternative to traditional counting methods^71^. Our approach is ideal for detecting distinct objects that may not be identical but have similar visual characteristics in images. We trained Deep Learning models to automate object detection with drone imagery. This software utilizes a modified version of the U-NET architecture^72^, a type of CNN, to enable instance segmentation without requiring a complex and data-intensive model such as Mask-R-CNN^73^. We created three detectors that specifically targeted calves and older elephants (age 1 + years old), and all elephants regardless of age (age 0 + years) (Fig. 6). This approach enabled us to train each detector separately, ensuring no confusion between the classes. We utilized drone orthomosaics for training the software by outlining polygons around each elephant. Approximately 10 elephants were used for each class during training. We trained each detector with 4000 steps. The accuracy of the binary classification model, the F1 score, was estimated as in^74^:

$$F1=\frac{2*Precision*Recall}{Precision+Recall}$$

In the context of this study, "precision" refers to the ratio of false positives (FP) and false negatives (FN) in the classifications. "Recall" is defined as TP/(TP + FN), where TP represents the true positive rate as determined by a human observer. The accuracy of the classifications was exceedingly high, with calves having a 94.14% accuracy rate, older elephants with 95.06%, and the entire elephant group with 90.75%. After running the detectors on the drone mosaics, each elephant was indicated with a polygon and data on its position (latitude and longitude), body area and perimeter were recorded.

We utilized the polygons classified by the ML for each elephant to determine body size. To automate the measurement of elephant length and width, we developed a custom-written R function (Fig. S3)^75^. Within this function, spatial polygons were plotted and analysed using the packages “sf” and “lwgeom” with further manipulation of the data using package “reshape2”^76,77^. To remove limbs from computation, polygons were smoothed by Gaussian Kernel regression with a bandwidth value of two, using the R-package “smoothr”^78^. To determine the length of an elephant from head to tail, we used the two furthest points on its polygon to represent these body parts. We divided the polygon into two-line segments at these points and constructed a line that followed the curvature of the polygon by using a set of points midway between corresponding coordinate points in each line segment. Accounting for the effect of smoothing, we added the distance between the farthest points in the smoothed polygon and the original polygon to the length of this curved line. This value represented the length of the elephant from head to tail, following the curvature of its body. All relevant code is available.

We also manually estimated the standard body length of elephants using classified polygons and mosaics to validate the automatic calculation. To do this, we measured the straight-line distance from the head to the tip of each elephant's tail^79,80^. We utilized 10 panels with known areas ranging from 1.2 to 7.5 m, which were photographed during 3 drone missions to calculate the error of body length measurements. While installing a radio collar on an elephant's neck, one individual from each group of elephants is physically measured for their body length. As an example, the results of the analysis show the elephant's body length was manually measured at 2.9 m, while the corresponding automatic measurement from a drone was 2.9 ± 0.58 m. The measurements taken by the drone closely matched the manually measured dimensions of the elephant's body length (F = 1875, p < 0.001, R^2^ = 0.94, y = 1.25x + 0.0800; Fig. S4).

### Estimation of population size

We consider a model for spatially replicated counts proposed by^38^, also known as N-mixture models. The objective of this model is to estimate the abundance of the species of interest without the need of individual identification, while the model can be easily extended for understanding the relationship between the abundance and site-specific covariates. Therefore, N-mixture model has been recently extended for modelling the aerial counts^51,81,82^. However, we note that some potential issues in applying N-mixture modeling to wildlife abundance have been highlighted, such as a lack of identifiable parameters when abundance, probability of detection, or the number of visits to survey sites is low^51,82,83^.

In this paper, we fit the aerial counts on the N-mixture model for estimating the abundance of the elephant. For clarity, we start by describing the notations for the model. Let ${n}_{jt}$ be the number of unique individuals detected at location $j$ at time $t$ for sampling occasions $t=1,\dots ,T$ and locations $j=1,\dots ,R$. We assume that within the sampling occasion $T$, the target population is closed i.e., no birth/death and migration/emigration is allowed, and individuals are detected independently over locations and times. The count data is assumed to follow a binomial distribution such that:

$${n}_{jt}\sim {\text{Binomial}}\left({N}_{j}, \psi \right),$$

where ${N}_{j}$ is the population size at location $j$ and $\psi$ is the detection probability. In this paper, we further assume the detection probability to be constant over time and space i.e., $\psi$ is the same for all $j$ and $t$ for simplicity. The realized total population ${N}_{total}$ is equal to the sum of the estimated population size for all locations $j$ such that ${N}_{total}=\sum_{j=1}^{R}{N}_{j}$.

To estimate the model parameters, a Bayesian approach is used for this case considering the sample size which is quite small. Therefore, we need to assign prior distributions to each parameter in the model to obtain the posterior densities of parameters. We assume a Poisson prior distribution for ${N}_{j}$ such that:

$${N}_{j}\sim {\text{Poisson}}\left(\lambda \right),$$

where $\lambda$ is the expected population size and $\lambda \sim {\text{Uniform}}\left(0, 100\right)$ with the upper limit is chosen such that, it does not affect the posterior distribution of $\lambda$ i.e., ${\text{Pr}}\left(\lambda =100\right)\approx 0$. For detection probability, a Beta prior is assumed such that $\psi \sim {\text{Beta}}\left(1, 1\right)$. ${\text{Beta}}\left(\mathrm{1,1}\right)$ is chosen to constrain the detection probability $\psi$ such that $\psi \in \left[0, 1\right]$. Note that^28^ suggested an alternative prior specification for $\lambda$ i.e., a gamma prior which is equivalent to the Negative-Binomial prior for ${N}_{j}$. However, the Poisson-Uniform prior seems to be sufficient for our case therefore we restrict the analysis for given priors. Finally, site-specific covariates can be incorporated into $\lambda$ and/or $\psi$ if such information is available. In this paper, we simply fit the simple model where $\lambda$ and $\psi$ are assumed to be invariant. Let ${\varvec{N}}=\left\{{N}_{j};j=1,\dots ,R\right\}$ and ${\varvec{n}}=\left\{{n}_{jt};j=1,\dots ,R;t=1,\dots ,T\right\}$, thus the joint posterior distribution over the model parameters is given by:

$$\pi \left({\varvec{N}}, \lambda ,\psi |{\varvec{n}}\right)\propto f\left({\varvec{n}}|p, {\varvec{N}}\right)p\left(\psi \right)p\left({\varvec{N}}|\lambda \right)p\left(\lambda \right),$$

where $$f\left({n}_{jt}|p, {N}_{j}\right)$$ is the joint likelihood function of the data and $$p\left(.\right)$$ denotes the prior distribution of the corresponding parameters defined earlier. We fit the count data of the elephant on the N-mixture model defined in Eq. ($$\pi \left({\varvec{N}}, \lambda ,\psi |{\varvec{n}}\right)\propto f\left({\varvec{n}}|p, {\varvec{N}}\right)p\left(\psi \right)p\left({\varvec{N}}|\lambda \right)p\left(\lambda \right)$$) using a Bayesian method. The count data was collected from the aerial survey using drones at 8 different locations ($$R=8)$$ (Fig. S5). The survey was conducted for four days resulting in $$T=4$$, hence the closure assumption is well satisfied. Note that we only include locations with at least one individual being detected during the observations. We run Markov chain Monte Carlo (MCMC) algorithm for 50,000 iterations following an initial 5000 burn-in for each algorithm using 3 separate and independent chains. The model was fitted using the R package rjags^84^. Next, The trace plot and the Brooks–Gelman–Rubin statistic for each parameter are obtained for convergence check of the MCMC samples^85^.

### Age prediction

We consider two models for age prediction of elephants in this paper: (i) Von Bertalanffy growth curve model, and (ii) generalized additive model (GAM). The growth curve derived by^86^ has been used for studying the growth phenomena on vertebrates including elephants^80,87^. The model is used to explain the growth curve of body sizes e.g., back length based on the individual ages^59,61,86,88^. Let ${L}_{i}$ be the body size (the total body length) of individual $i$ and ${t}_{i}$ denote the age of associated individuals for individuals $i=1, \dots , n$. The von Bertalanffy growth function has a form of:

$${L}_{i}={L}_{\infty }-\left({L}_{\infty }-{L}_{0}\right){\text{exp}}\left(-K{t}_{i}\right),$$

where ${L}_{\infty }$ is the asymptotic total body length, ${L}_{0}$ is the body length at birth and $K$ is a rate constant. Note that $\left\{{L}_{\infty }, {L}_{0}, K\right\}$ are growth parameters to be estimated.

The second model we consider for predicting the age of elephants is generalized additive model (GAM). Under the GAM, we assume that age, a dependent variable, is a function of an unknown smooth function. The form of the generalized additive model for age can be written mathematically as:

$$g\left({t}_{i}\right)={\beta }_{0}+s\left({L}_{i}\right)+{\epsilon }_{i},$$

for $i=1,\dots ,n$ where ${\beta }_{0}$ is a constant, $g\left(.\right)$ is a link function to be chosen; and ${\epsilon }_{i}$ is a zero mean error term with variance ${\sigma }^{2}$. The function $s\left(.\right)$ denotes an unknown smooth function. In this work, we consider a basis expansion to approximate $s\left(.\right)$ such that $s\left({L}_{i}\right)=\sum_{r=1}^{m}{\beta }_{r}{b}_{r}\left({L}_{i}\right),$ where ${b}_{r}\left({L}_{i}\right)$ is a B-spline basis function and ${\beta }_{r}$ are coefficients to be estimated^60^. The link function $g\left(.\right)$ is chosen such that it constrains the age into the interval $\left[0, \infty \right]$. Therefore, a Gamma distribution is assigned to fit the model to the age given the body length since the function is defined for all positive real numbers, and the log-link function is chosen such that $g\equiv$ log. Note that a Gaussian distribution with a log-link function may be an alternative distributional form for modelling age with a constant variance. However, we prefer a Gamma distribution where the variance is not necessarily constant i.e., we expect to have smaller uncertainties for smaller body length. We also note that the inverse of the von Bertalanffy growth function can be written as a linear function of the body length^59^ such that:

$$t=\frac{1}{K}{\text{log}}\left({L}_{\infty }-{L}_{0}\right)-\frac{1}{K}{\text{log}}\left({L}_{\infty }-{L}_{i}\right),$$

which is equivalent to the GAM equation with ${\beta }_{0}=\frac{1}{K}{\text{log}}\left({L}_{\infty }-{L}_{0}\right)$ and $s\left({L}_{i}\right)=-\frac{1}{K}{\text{log}}\left({L}_{\infty }-{L}_{i}\right)$. Fitting the inverse of von Bertalanffy growth equation to the age assuming a Gamma distribution with a log-link function is somehow similar to the initial GAM but a different function in $s\left({L}_{i}\right)$. Thus, we consider GAM is an appropriate and a reasonable approximation to the original von Bertalanffy growth function when the main objective is the prediction not the relationship itself.

Note that drone photogrammetry has been used to predict the age of hippopotamuses in nature using multiple imputation statistical analyses^61^. However, the conclusions of such data-driven models are limited without ground-truthing the findings with direct measurements from morphometric data collected in the field. Considering the difficulties in obtaining total body length data and other body measurements of Sumatran elephants in nature, validation data is needed in the form of appropriate morphometric measurements for age and sex, commonly available in ex-situ locations such as zoos. Therefore, we collect an additional dataset from the zoo corresponding to body measurements and their associated ages of 23 individuals with a range of age between 1 and 40 years old. We treated the additional dataset from the zoo as training data to obtain the parameter estimates of the von Bertalanffy growth model and GAM. The growth parameters obtained are used to derive the inverse of the von Bertalanffy model to back-calculate age from the total body length^59^. We use the R package FSA to obtain the maximum likelihood estimates of the von Bertalanffy growth parameters^89^ where the 95% confidence interval of corresponding parameters are derived using a nonparametric bootstrap. For GAM, we fit the model by setting age as a dependent variable and the body length as a predictor assuming a Gamma model with a log-link function using R package mgcv^90^. Parameter estimates from both the Von Bertalanffy growth model and GAM are used for comparing the age prediction for given body lengths while assessing the uncertainties e.g., 95% prediction intervals of the prediction. The primary objective of building such models is to estimate the age of elephants from the aerial survey data i.e., predicting individual ages based on the total body length estimated from the snapshot of aerial survey (Fig. S5). We further classify the age group into four classes according to their maturity, i.e., calf (0–1 year), juvenile (1–5 years), sub-adult (5–15 years) and adult (> 15 years)^80^.

### Ethics declaration

Ethical review and approval were not required for this study. We use a non-invasive approach in collecting wild animal data. No live animals were used for this research, and no humans were part of this study. All work was conducted in accordance with Animal Welfare guidelines and received permits and support from the Ministry of Environment and Forestry, the Regional Government of Jambi Province and Forest and Non-Forest Utilization Concessions.

### Supplementary Information

Supplementary Information.

## Data Availability

The datasets used and/or analyzed during the current study are available from the corresponding author on request.
