# Supplementary material for: The first use of a photogrammetry drone to estimate population abundance and predict age structure of threatened Sumatran elephants
Source: Sci Rep. 2023 Dec 3;13:21311. doi: 10.1038/s41598-023-48635-y (PMC10693614; doi:10.1038/s41598-023-48635-y)
Supplement: Supplementary file 1 — Supplementary Information. [file 41598_2023_48635_MOESM1_ESM.docx]

**Supplementary Material**

**Table S1**. Age prediction of the elephants and associated 95% lower and upper confidence limits (LCL and UCL) using two different models: Von Bertalanffy growth model and GAM fitted on 23 known-age individuals.

| **Body Length** | **von Bertalanffy Growth model** | | | **GAM** | | |
| --- | --- | --- | --- | --- | --- | --- |
|  | **Estimated Age** | **LCL** | **UCL** | **Estimated Age** | **LCL** | **UCL** |
| 130 | 0 | 0 | 6 | 2 | 1 | 2 |
| 135 | 0 | 0 | 10 | 2 | 1 | 3 |
| 140 | 1 | 0 | 14 | 2 | 2 | 3 |
| 145 | 1 | 0 | 17 | 3 | 2 | 3 |
| 150 | 1 | 0 | 21 | 3 | 3 | 4 |
| 155 | 2 | 0 | 25 | 4 | 3 | 4 |
| 160 | 2 | 0 | 30 | 4 | 4 | 5 |
| 165 | 2 | 0 | 34 | 5 | 4 | 6 |
| 170 | 3 | 0 | 38 | 6 | 5 | 7 |
| 175 | 3 | 0 | 43 | 7 | 6 | 8 |
| 180 | 4 | 0 | 48 | 8 | 7 | 10 |
| 185 | 4 | 0 | 53 | 9 | 8 | 11 |
| 190 | 4 | 0 | 58 | 11 | 9 | 13 |
| 195 | 5 | 0 | 63 | 12 | 10 | 14 |
| 200 | 5 | 0 | 68 | 13 | 11 | 16 |
| 205 | 6 | 0 | 74 | 14 | 12 | 17 |
| 210 | 6 | 0 | 80 | 15 | 13 | 18 |
| 215 | 7 | 0 | 86 | 17 | 14 | 20 |
| 220 | 7 | 0 | 92 | 18 | 15 | 21 |
| 225 | 8 | 0 | 99 | 19 | 16 | 22 |
| 230 | 8 | 0 | 106 | 20 | 17 | 24 |
| 235 | 9 | 0 | 114 | 21 | 18 | 25 |
| 240 | 9 | 1 | 121 | 22 | 18 | 27 |
| 245 | 10 | 1 | 130 | 23 | 19 | 28 |
| 250 | 10 | 1 | 138 | 24 | 20 | 30 |
| 255 | 11 | 1 | 148 | 25 | 20 | 31 |
| 260 | 11 | 1 | 158 | 26 | 21 | 32 |
| 265 | 12 | 1 | 168 | 27 | 22 | 34 |
| 270 | 12 | 1 | 180 | 28 | 22 | 35 |
| 275 | 13 | 1 | 192 | 29 | 23 | 36 |
| 280 | 13 | 1 | 206 | 30 | 24 | 38 |
| 285 | 14 | 1 | 221 | 31 | 25 | 39 |
| 290 | 15 | 1 | 238 | 32 | 26 | 40 |
| 295 | 15 | 1 | 257 | 33 | 27 | 41 |
| 300 | 16 | 1 | 279 | 34 | 28 | 42 |
| 305 | 16 | 1 | 305 | 35 | 29 | 43 |
| 310 | 17 | 1 | 337 | 36 | 29 | 44 |
| 315 | 18 | 1 | 377 | 37 | 30 | 46 |
| 320 | 18 | 1 | 434 | 38 | 30 | 49 |
| 325 | 19 | 1 | 528 | 39 | 29 | 52 |
| 330 | 20 | 1 | 892 | 40 | 28 | 57 |


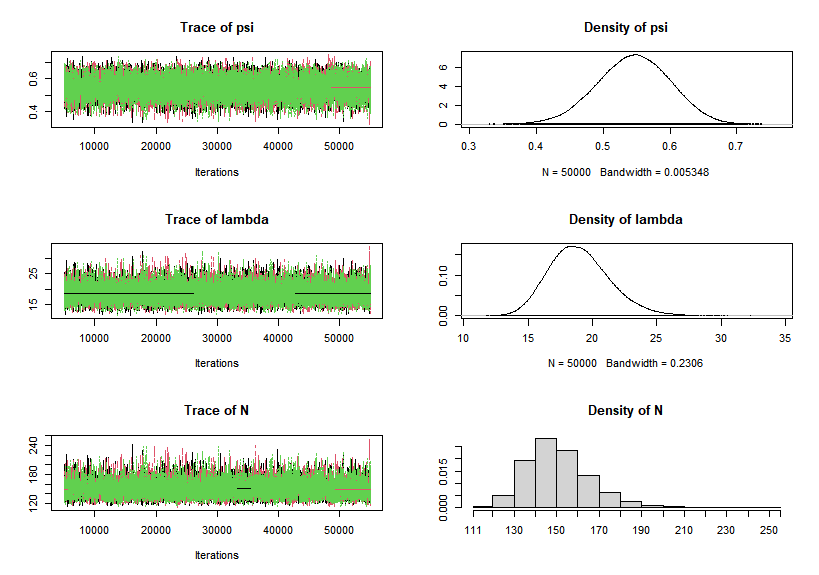


**Figure S1**. Posterior distributions of the MCMC samples of monitored parameters for N-mixture model: $\psi, \lambda$ and $N_{total}$ for 50,000 iterations of three independent chains.


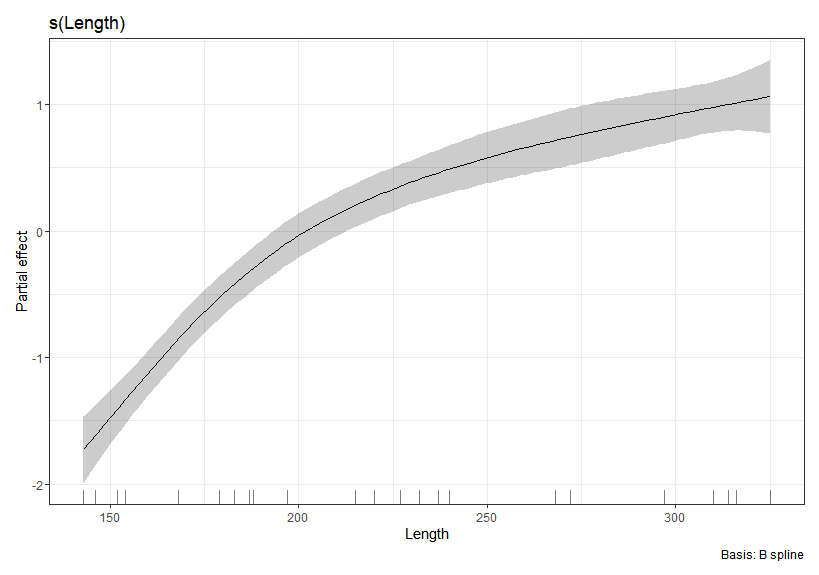


**Figure S2**. The partial effect of body length on the smooth terms (B-spline) in the GAM model.


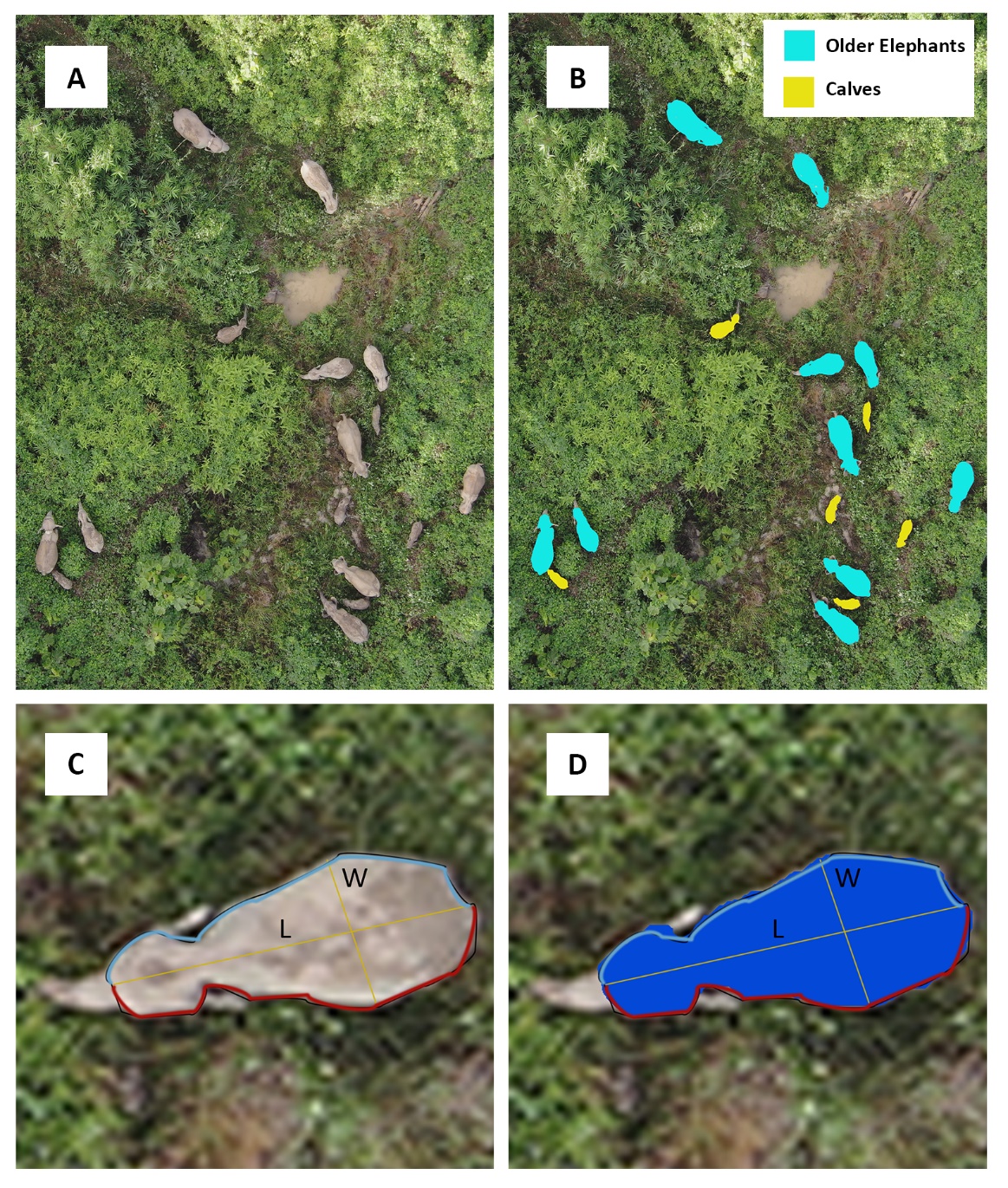


**Figure S3**. On November 6, 2022, machine learning detectors were used to identify Sumatran elephants in Bukit Tigapuluh Landscape, Indonesia. (A) A drone orthomosaic map is composed of many overlapping photos. (B) Yellow and blue polygons indicate calves and older elephants identiﬁed by machine learning, (C, D) automatic measurement of body lengths (L) and widths (W) describe elephant shape. Polygons were smoothed and split into two-line segments (red and light blue lines). Coordinate points midway between these segments were used to construct a line representing the position of the spine. To account for the effect of smoothing, the distance between the smooth and unsmoothed polygon was added to the length of this line to calculate elephant length (long orange line). Widths were calculated based on the maximum distance between the curved line and the smoothed polygon (short orange line).


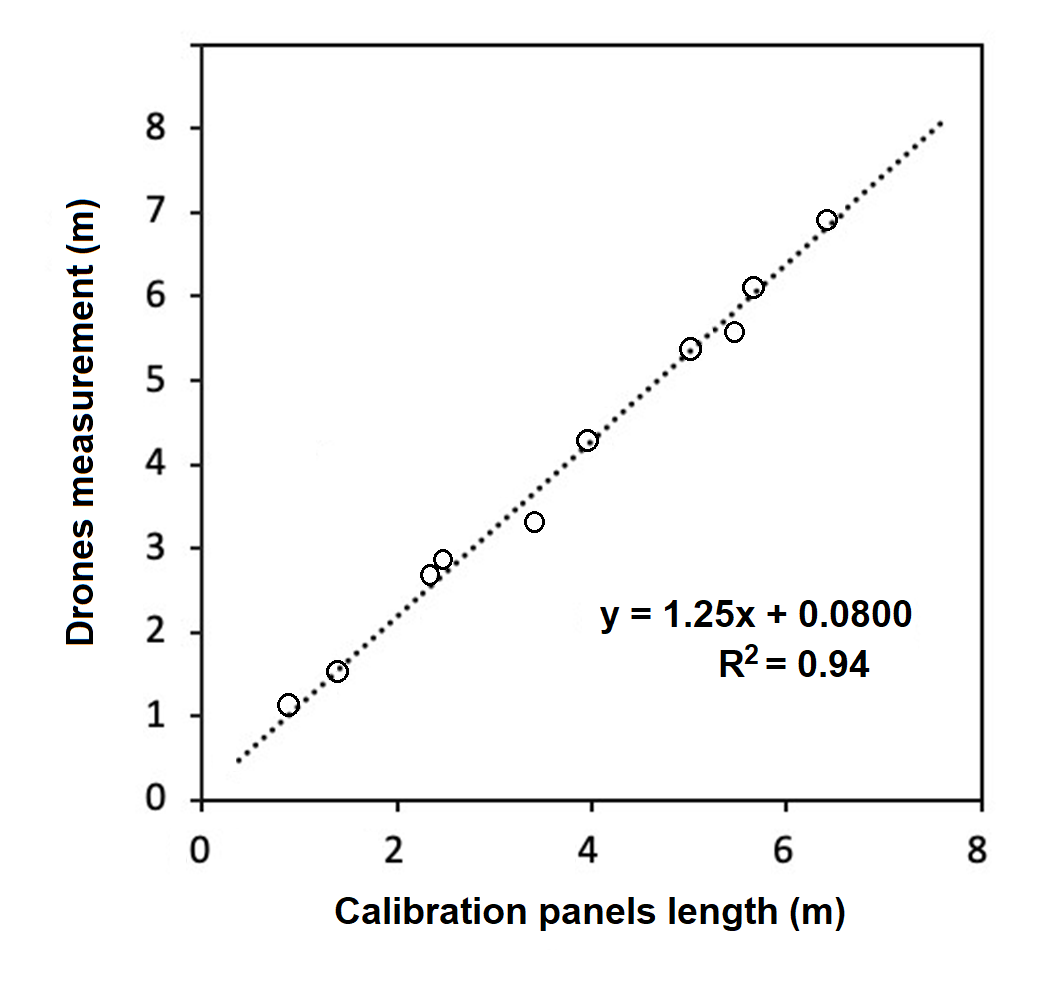


**Figure S4**. Evaluation of the accuracy of automatic area measurements with the drone from orthomosaic photo maps. 10 panels with known areas from 1.2 to 7.5 m were used to compare with the area measured by the Machine Learning in the drone mosaics.


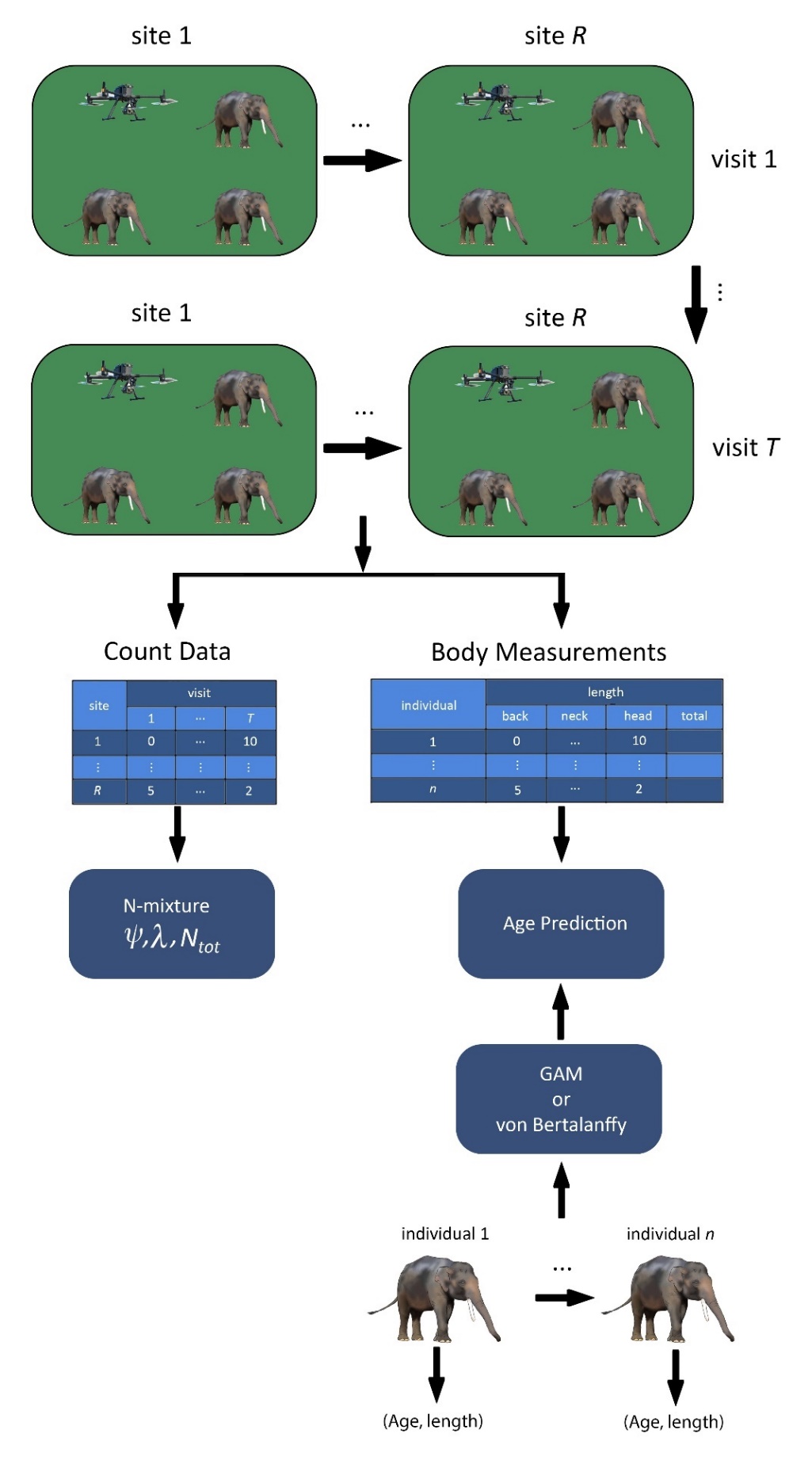


**Figure S5**. Diagram of the survey methods and workflow tested in the study. Drone missions were pre-programmed with automatic flight. Drones were flown repeatedly at each elephant finding location. The number of individual findings per location is used to estimate population density with the N-mixture model, while the results of body size measurements with machine learning are used to predict the age of each individual elephant.
